# Hilar Parenchymal Oversew: a novel technique for robotic partial nephrectomy hilar tumor renorrhaphy

**DOI:** 10.1590/S1677-5538.IBJU.2017.0049

**Published:** 2018

**Authors:** Jaya Sai S. Chavali, Ryan Nelson, Matthew J. Maurice, Onder Kara, Pascal Mouracade, Julien Dagenais, Jeremy Reese, Pilar Bayona, Georges-Pascal Haber, Robert J. Stein

**Affiliations:** 1 Department of Urology, Glickman Urological and Kidney Institute, Cleveland Clinic, Cleveland, OH, USA

## Abstract

**Introduction:**

A renorrhaphy technique which is effective for hemostasis but does not place undue tension on the branch vessels of the renal sinus remains one of the challenging steps after hilar tumor resection during robotic partial nephrectomy (RPN). The published V-hilar suture (VHS) technique is one option for reconstruction after an RPN involving the hilum. The objective of this video is to show a novel renorrhaphy technique, Hilar Parenchymal Oversew that has been effective for such cases.

**Materials and Methods:**

We present two cases of RPN for renal hilar tumors. The first case depicts use of the VHS renorrhaphy technique for a tumor that abuts the renal hilum along 20% of its diameter. The second case demonstrates tumor resection and reconstruction for a tumor that has >50% involvement of the hilum along its diameter. After tumor resection, individual sinus vessels can be selectively oversewn with 2-0 Vicryl suture on SH needle. The remaining exposed parenchyma is controlled using the Hilar Parenchymal Oversew technique with a #0 Vicryl on CT-1 needle.

**Results:**

For the Hilar Parenchymal Oversew surgery operative time was 225 min, estimated blood loss was 140 ml, warm ischemia time was 19 minutes, and there were no intraoperative complications. Pathology was consistent with clear cell renal cancer with negative margins.

**Conclusion:**

Robotic partial nephrectomy with the Hilar Parenchymal Oversew technique is a good alternative to VHS renorrhaphy in the management of renal hilar tumors “bulging” into the renal sinus with >50% of the tumor diameter abutting the hilum.

## ARTICLE INFO

**Available at: http://www.intbrazjurol.com.br/video-section/20170049-Chavali_et_al**

**Int Braz J Urol. 2018; 44 (Video #1): 199-199**

